# Stroking modulates noxious-evoked brain activity in human infants

**DOI:** 10.1016/j.cub.2018.11.014

**Published:** 2018-12-17

**Authors:** Deniz Gursul, Sezgi Goksan, Caroline Hartley, Gabriela Schmidt Mellado, Fiona Moultrie, Amy Hoskin, Eleri Adams, Gareth Hathway, Susannah Walker, Francis McGlone, Rebeccah Slater

**Affiliations:** 1 Department of Paediatrics, University of Oxford, Oxford, OX3 9DU, UK; 2 School of Life Sciences, University of Nottingham, Nottingham, NG7 2UH, UK; 3 Department of Natural Sciences and Psychology, Liverpool John Moores University, Liverpool, L3 3AF, UK; 4 Institute of Psychology, Health and Society, University of Liverpool, Liverpool, L69 3BX, UK

## Abstract

A subclass of C fibre sensory neurons found in hairy skin are activated by gentle touch [1] and respond optimally to stroking at ∼1–10 cm/s, serving a protective function by promoting affiliative behaviours. In adult humans, stimulation of these C-tactile (CT) afferents is pleasant, and can reduce pain perception [2]. Touch-based techniques, such as infant massage and kangaroo care, are designed to comfort infants during procedures, and a modest reduction in pain-related behavioural and physiological responses has been observed in some studies [3]. Here, we investigated whether touch can reduce noxious-evoked brain activity. We demonstrate that stroking (at 3 cm/s) prior to an experimental noxious stimulus or clinical heel lance can attenuate noxious-evoked brain activity in infants. CT fibres may represent a biological target for non-pharmacological interventions that modulate pain in early life.

## Teaser

Gursul *et al.* find that gentle stroking of the skin at a frequency that stimulates C-fibre sensory neurons relieves pain in infants.

## Main Text

Noxious-evoked brain activity in infants is similar to that observed when adults experience pain [4], providing an objective method of assessing pain-relieving interventions [5]. We hypothesised that touch which optimally activates CT fibres in adults would reduce noxious-evoked brain activity measured using electroencephalography (EEG). In 30 term infants, we applied CT-optimal touch (brush velocity 3 cm/s), CT non-optimal touch (brush velocity 30 cm/s), and a no-touch control in 5-second blocks before each acute experimental noxious stimulus (128 mN PinPrick™ Stimulation, MRC Systems) in a train of nine stimuli (Study 1). We then tested whether CT-optimal stimulation reduced noxious-evoked responses in a clinical context in an independent sample of infants (Study 2).

**Figure 1:**
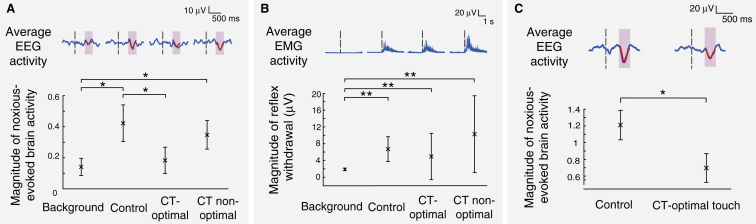
CT-optimal touch reduces noxious-evoked brain activity following experimental and clinically required noxious stimulation in infants. (A) Top: average background EEG activity, and responses to the first experimental noxious stimulus in the no-touch control, CT-optimal (∼3 cm/s), and CT non-optimal (∼30 cm/s) touch conditions (number of infants = 30). Traces are Woody filtered and shown overlaid with the template of noxious-evoked brain activity in red. This template [5] was used to calculate the magnitude of the noxious-evoked brain activity within each individual trial — see experimental procedures in Supplemental Information. For reference, a magnitude of 1 represents the average evoked response to a heel lance in a group of term-aged infants. Black dashed lines indicate the point of noxious stimulation; pink shading indicates the time window of interest for noxious-evoked brain activity. Bottom: the magnitude of the noxious-evoked brain activity in the background period and in response to experimental noxious stimulation following the no-touch control, CT-optimal, and CT non-optimal touch conditions in the first trial. (B) Top: average limb reflex withdrawal response to the first experimental noxious stimulus in each condition. Bottom: the magnitude of the limb reflex withdrawal quantified using root mean square (RMS) following the first noxious stimulus. (C) Top: average EEG response in no-touch control and the CT-optimal touch conditions following a clinically required heel lance (number of infants = 16 in each group). Traces are Woody filtered and shown overlaid with the template of noxious-evoked brain activity in red. Black dashed lines indicate the time of the heel lance; pink shading indicates the time window of interest for noxious-evoked brain activity. Bottom: CT-optimal touch significantly reduced the magnitude of the noxious-evoked brain activity. Error bars indicate mean ± standard error; ^∗^ indicates p < 0.05; ^∗∗^ indicates p < 0.01.

In Study 1, CT-optimal touch significantly reduced noxious-evoked brain activity following the first acute experimental noxious stimulus (p = 0.029, linear mixed effects analysis; Figure 1A). In contrast, CT non-optimal touch did not significantly reduce noxious-evoked brain activity (p = 0.57, linear mixed effects analysis; Figure 1A), demonstrating the specificity of the response to lower velocity stroking. In the CT-optimal condition, the attenuation of noxious-evoked brain activity was not maintained across repeated stimuli (p = 0.62, all CT-optimal trials compared with control, linear mixed effects analysis). The magnitude of noxious-evoked brain activity following the first noxious stimulus in the CT-optimal condition was significantly lower than the magnitude of the noxious-evoked brain activity in subsequent trials (p = 0.019; Figures S1A and S2A in Supplemental Information, published with this article online), consistent with CT fibre fatigue [6]. There was, however, no suggestion that CT-optimal (p = 0.20, Wilcoxon signed rank test, first trial compared with first trial of control) or non-optimal (p = 0.40) tactile stimulation altered limb reflex withdrawal activity (Figures 1B and S1A). The magnitude of the noxious-evoked brain activity following the first CT-optimal brush stimulation was approximately 60% less than the magnitude of the activity evoked in the no-brush condition. This is a similar effect size to that reported in a previous study of topical local anaesthetic [5]. However, the effect of topical anaesthethic in this study is likely to have been conservative due to the limited time allowed for absorption (30 minutes) prior to application of the noxious stimuli.

To test whether CT-optimal tactile stimulation is clinically effective we applied it for 10 s prior to clinical heel lancing for blood collection in an independent sample of 32 infants; 16 infants received CT-optimal stimulation and 16 were aged-matched controls (Study 2). CT-optimal stimulation significantly reduced the magnitude of noxious-evoked brain activity compared with age-matched controls who were not touched prior to lancing (p = 0.045, two-sided t-test; Figure 1C). A 40% reduction in the magnitude of noxious-evoked brain activity following the heel lance was observed when the brush stimulation was applied. Interestingly, one infant required two heel lances (∼4 minutes apart) and CT-optimal touch was performed before each lance. The magnitude of noxious-evoked brain activity was similar, suggesting that CT-optimal touch stimulation was equally influential for each procedure (Figure S2B). This is in contrast to our results from Study 1, where the experimental noxious stimuli were applied in relatively quick succession and CT-fibre fatigue is likely to have contributed to the observed lack of efficacy of the brush intervention. These findings suggest that the timing of the touch intervention relative to the noxious stimulation needs to be carefully considered if this technique is to be developed for therapeutic use, and further work is required to establish the optimal timing.

Consistent with Study 1, the magnitude of the reflex withdrawal was not significantly different between groups (p = 1, Mann-Whitney U-test; Figure S1B). While noxious-evoked reflexes and brain activity are correlated in term infants [7], these data suggest that CT-optimal tactile stimulation may disrupt this relationship. Albeit, the study was not powered to see a significant reduction in reflex withdrawal activity. Recently, we have shown that resting state functional connectivity between brain areas involved in endogenous pain modulation influences the magnitude of noxious-evoked brain activity [4]. It is possible that CT-optimal tactile stimulation may influence noxious-evoked brain activity by engaging this maturing pain modulatory system. Inhibition of spinal reflexes is, however, not observed, indicating that, consistent with rodent studies, communication of descending inhibitory influences via spinal projections may be immature [8]. Concomitant maturation of brain activity and reflex withdrawal activity occurs during preterm development [9], and by term descending modulatory centres may begin to exert inhibitory influences. However, evidence here suggests that this system is not fully mature, or at least not engaged by CT-optimal tactile stimulation.

We also examined infants’ behavioural responses in Study 2 by recording the duration of pain-related facial expressions (see Supplemental Information). A similar proportion of infants exhibited facial grimacing following heel lancing in both groups (12/16 infants in the CT-optimal touch group; 9/14 in the control group). However, the duration was almost 50% shorter in infants who received CT-optimal stimulation (median duration (lower quartile, upper quartile) = 7 s (4, 13) than in the control group = 13 s (9, 14); Figure S1C; p = 0.30, Mann-Whitney U-test). The study was not powered to investigate this behavioural effect, and further investigation is warranted. This finding is consistent with some observations that tactile stimulation, such as infant massage, can reduce behavioural pain scores [3]. It is plausible that CT fibre stimulation may represent a neurophysiological mechanism underlying the efficacy of these interventions.

Microneurography has facilitated identification of CT fibres in adults, but the safe use of this invasive technique has not been established in infants [1]. Our results nevertheless suggest that tactile stimulation, at a velocity that activates CT fibres in adults, can modulate noxious-evoked brain activity in infants. Social touch is important for parent–infant bonding and parents instinctively stroke infants at a CT-optimal velocity [10]. Further research is needed to ascertain whether this simple tactile intervention is effective in modulating pain in the context of other clinical procedures and in preterm infants. Better understanding of the role of CT fibres in early life may lead to the development of neurobiologically driven interventions to optimise pain management in neonatal care.

## Supplemental Information

Document S1. Two Figures and Experimental Procedures
