# Supplementary material for: Stroking modulates noxious-evoked brain activity in human infants
Source: Curr Biol. 2018 Dec 17;28(24):R1380–1. doi: 10.1016/j.cub.2018.11.014 (PMC6303187; doi:10.1016/j.cub.2018.11.014)
Supplement: Document S1. Two Figures and Experimental Procedures [file mmc1.pdf]

Supplemental Information: Stroking modulates noxious-evoked brain activity in human infants

Deniz Gursul, Sezgi Goksan, Caroline Hartley, Gabriela Schmidt Mellado, Fiona Moultrie, Amy Hoskin, Eleri Adams, Gareth Hathway, Susannah Walker, Francis McGlone and Rebeccah Slater.

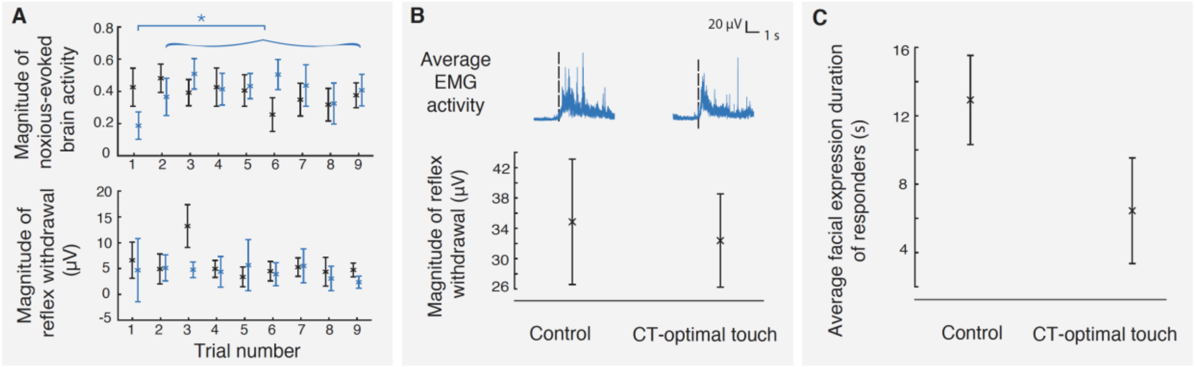

Figure S1. Related to Figure 1 and Figure S2. (A) Magnitude of noxious-evoked brain activity and reflex withdrawal activity with trial number in the no touch and CT-optimal touch conditions, (B) Average reflex withdrawal activity in response to heel lancing, (C) Average duration of facial grimacing following heel lancing.

(A) Top: the magnitude of the noxious-evoked brain activity to the experimental noxious stimulation plotted per trial following no touch (control, black) and CT-optimal touch (blue) conditions. In the CT-optimal touch condition, the magnitude of noxious-evoked brain activity in response to the first noxious stimulus was significantly lower than the magnitude of noxious-evoked brain activity in response to subsequent trials. Bottom: the magnitude of the limb reflex withdrawal (RMS) to the experimental noxious stimulus per trial following no-touch control (black) and CT-optimal touch (blue) conditions. Error bars indicate mean (brain activity) or median (limb reflex withdrawal) ± standard error. (B) Top: average limb reflex withdrawal responses in no-touch control and the CT-optimal touch conditions following a clinically required heel lance. Bottom: the magnitude of the limb reflex withdrawal (RMS) following heel lancing in the two groups. (C) The average duration of noxious-evoked facial expression change following a clinically required heel lance in the no-touch control and the CT-optimal touch conditions ($p = 0.30$). Error bars indicate mean ± standard error. * indicates $p < 0.05$.

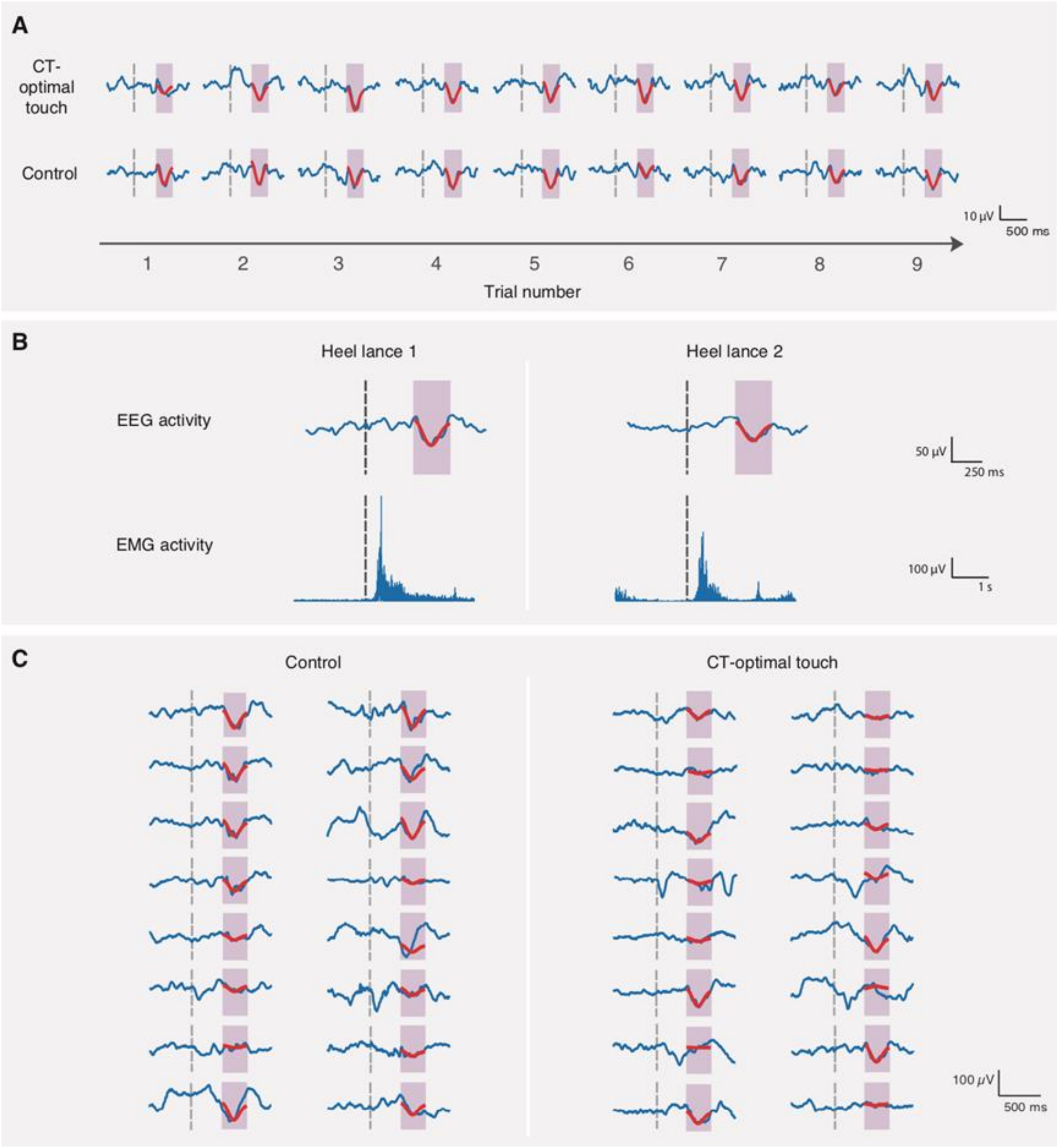

Figure S2. Related to Figure 1 and Figure S1 (A) Noxious-evoked brain activity with trial number in the CT-optimal touch condition and the control condition, (B) CT-optimal touch prior to two heel lances in the same infant results in similar magnitude noxious-evoked brain activity and reflex withdrawal, (C) Individual noxious-evoked brain activity following heel lance stimulation in all infants (Study 2).

(A) The magnitude of the average noxious-evoked brain activity in the CT-optimal touch condition is dependent on trial number. In contrast, the magnitude of the average noxious-evoked brain activity in the control condition is not dependent on trial number. Group average EEG activity is shown, overlaid with the template of noxious-evoked brain activity in red. The template typically explains about 30% of the variance of the signal in the EEG data [S1-S3]. The goodness-of-fit of the data to the template was calculated in response to the first stimulus in the CT-optimal touch condition (goodness-of-fit = 0.16 $\pm$ 0.45, mean $\pm$ standard deviation of the correlation with the template) and compared with the first stimulus in the control condition (goodness-of-fit = 0.34 $\pm$ 0.38). This did not represent a significant difference (p=0.17). (B) One infant in Study 2 clinically required two heel lances, and received CT-optimal touch prior to both. The heel lances were performed approximately 4 minutes apart. The EEG and EMG activity following each individual heel lance is shown. (C) Individual EEG waveforms are shown for all infants. All EEG waveforms shown in this figure are Woody filtered and overlaid with the template of noxious-evoked brain activity in red. Dashed lines indicate the point of noxious stimulation; pink shaded boxes indicate the time window of interest for noxious-evoked brain activity (400-700 ms).

Supplemental Experimental Procedures

Participants

Infants were recruited from the Maternity Unit at the John Radcliffe Hospital, Oxford University Hospitals National Health Service Foundation Trust, UK. Infants were eligible for inclusion if they were term, clinically stable, had no history of neurological problems, and were not receiving analgesics. Infant demographic characteristics are described in the table below.

	Study 1	Study 2	
		CT-optimal touch	Control
Number of infants	30	16	16
Gestational age at study (weeks)	40.0 (39.0, 41.0)	39.9 (38.0, 41.7)	39.8 (37.9, 41.3)
Postnatal age at study (days)	2 (1, 3)	5 (3.8, 5)	2.5 (1, 4)
Birthweight (g)	3557 (604)	3391 (577)	3685 (659)
Number of males	14 (46.7 %)	10 (62.5 %)	5 (31.3 %)
Number born by normal vaginal delivery	14 (46.7 %)	10 (62.5 %)	5 (31.3 %)
Average Apgar score at 1 minute	8.7 (1.5)	8.6 (2.6)	8.2 (1.8)

Table of infant demographics.

Values given are mean (standard deviation), median (lower quartile, upper quartile) or number (percentage).

Ethics

Ethical approval was obtained from the National Research Ethics Service (reference 12/SC/0447), and informed written parental consent was obtained prior to studying each infant. Studies were carried out in accordance with the Declaration of Helsinki and good clinical practice guidelines.

Sample size

A total of 33 infants were included in Study 1. Three infants were excluded due to infant restlessness, illness or technical difficulties, leaving 30 infants included in the analysis. Based on the results of Study 1, a power calculation was performed to determine the sample size needed for Study 2. In Study 1 an approximate 60% reduction in the magnitude of noxious-evoked brain activity in response to the first application of the noxious stimulus was observed following CT-optimal touch compared with the no touch condition. In an independent sample of infants receiving a heel lance the average magnitude of the noxious-evoked brain activity was 1 ± 0.9 (mean ± standard deviation) [S1]. With an age-matched sample, a sample size of 32, with 16 infants

receiving CT-optimal touch, would be required to observe a reduction of this effect size with 80% power at a significance level of 0.05. We recruited 20 infants (inflated sample size to account for missing data) to receive CT-optimal touch before a clinically necessary heel lance. 4 infants were removed from the analysis due to artefacts. 16 age-matched infants, who had not received touch stimulation prior to heel lancing, were selected from a group of infants whose heel lance responses had previously been recorded as part of other research studies. Infants in the control group were selected to provide the best pairwise match to minimise differences in the gestational age and postnatal age compared with the CT-optimal touch group.

Study design overview

Data from 69 term infants were analysed across two studies. Electrophysiological brain activity was recorded in response to noxious stimulation and we investigated whether CT-optimal touch prior to the noxious stimulation reduced the magnitude of the noxious-evoked brain activity. In Study 1, CT-optimal touch, CT non-optimal touch and no touch were applied prior to an experimental noxious stimulus. To confirm whether the results were applicable to a clinically relevant procedure, in Study 2 CT-optimal touch was applied prior to a medically required heel lance in an independent sample of infants.

Experimental Design

Study 1

Experimental noxious stimuli (force=128 mN, PinPrick™, MRC Systems, Germany) were applied to the heel in 3 blocks, with approximately 9 stimuli per block. Noxious stimulation with a force of 128 mN has been applied to term infants in previous studies, and is not associated with behavioural distress or tissue damage [S2, S4]. The noxious stimulation was preceded by either CT-optimal, CT non-optimal, or no touch in a randomised order. The touch stimulus was brush stimulation (SENSELAb™ Brush-05, Somedic.com, Sweden) applied for 5 seconds across approximately 10 cm of the lower leg ipsilateral to the heel receiving noxious stimulation. In the CT-optimal touch condition, the brush stimulation was applied at a velocity of approximately 3 cm/s, and in the CT non-optimal touch condition it was applied at a velocity of approximately 30 cm/s. The duration of the brush stimulation was pragmatically chosen and guided by previous studies [S5]. A primary consideration was to ensure that the total duration of the study was well tolerated by the infants. The experimenter was cued to apply the brushing velocity and noxious stimuli by following a computer visualisation coded using PsychoPy. There was an inter-stimulus interval of approximately 1 second between the end of the brush stimulation and the experimental noxious stimulus, and a minimum inter-trial interval of 10 s between noxious stimuli. The inter-trial interval was extended to allow the infant to settle if necessary. In the majority of cases (92%) all 9 stimuli were applied per block. In 6 infants excessive infant limb movement meant some cued stimuli were not applied. The mean number of stimuli considered in the analysis was 8.7 ± 1.3 per block.

Study 2
Infants were included if they required a heel lance as part of their routine clinical care. Heel lances (BD Microtainer Quikheel Infant Lancet, Becton, Dickinson and Company) were performed on the medial or lateral plantar surface of the heel, and the foot chosen was based on clinical judgment. In 20 infants, CT-optimal touch (3 cm/s) was applied for approximately 10 seconds prior to the heel lance. The duration of the brush stimulation was increased to 10 seconds as this was consistent with previous studies [S6, S7]. As a single heel lance was usually required the overall duration of Study 2 was substantially shorter than Study 1, allowing a longer stimulation period. Following rejections due to artefact, 16 infants were included in the analysis. There was an inter-stimulus interval of approximately 1 second between the end of the touch and heel lancing, and touch stimulation was applied to the lower leg ipsilateral to the heel receiving the lance. In the control infants (n=16), no touch was applied prior to the heel lance.

Recordings

Electrophysiological activity from DC to 400 Hz was acquired with a SynAmps RT 64-channel EEG/EP system (Compumedics Neuroscan). Activity was recorded with a sampling rate of 2000 Hz using CURRYscan7 neuroimaging suite (Compumedics Neuroscan). EEG electrodes were placed at Cz, CPz, C3, C4, Oz, FCz, T3, T4, with reference at Fz and ground at FPz, according to the modified international 10-20 system. The reference electrode was positioned at Fz because this site is minimally affected by muscle artefacts and located sufficiently distal to the central recording electrodes to ensure that noxious-evoked activity can be reliably recorded [S1-S3]. Preparation gel (Nuprep gel, D.O. Weaver and Co.) and conductive paste (Elefix EEG paste, Nihon Kohden) were used to optimise electrode contact with the scalp. EMG was recorded with bipolar electrodes placed on the biceps femoris of the leg.

The experimental noxious stimuli were time-locked to the EEG recordings using a high-speed camera (220 frames per second; Firefly MV, Point Grey Research Inc.) in 15 infants, and a contact trigger device (MRC Systems) in the other 15 infants, which were directly linked to the recordings at the time of acquisition. Video recordings were reviewed post-acquisition and the time of stimulation was manually event-marked as the point where the barrel of the stimulator was first depressed [S8]. In Study 2, heel lance stimuli were time-locked to the EEG recordings using an event detection interface as previously described [S9].

In Study 2 facial expressions were recorded using a video camera, and the point of the heel lance was manually time-locked to the recordings by the experimenter activating an LED light at the point of stimulation.

Analysis

EEG
For both studies, EEG signals were filtered 0.5 – 30 Hz, with a notch filter at 50 Hz. Signals were extracted in 1500 ms epochs with 500 ms before the stimulus, and baseline corrected to the pre-stimulus mean. Individual epochs were rejected if they contained artefact such as gross movement artefact, or if there was movement in the baseline period. In Study 1, considering the response to the first trial in each block, data

from 8 infants in the CT-optimal touch condition, 10 in the CT non-optimal touch condition, and 10 in the no touch condition were rejected.

A previously defined and validated template of noxious-evoked brain activity was used to calculate the magnitude of the noxious-evoked brain activity for each individual trial at the Cz electrode in the time window 400-700 ms after the stimulus [S1]. The template was designed to be projected onto an individual infant's EEG activity in order to quantify the magnitude of noxious-evoked brain activity within a pre-specified time-window. The magnitude of the noxious-evoked activity is defined as the weight of the template after it is projected onto the infant's EEG activity to best fit the data. For reference, a magnitude of 1 represents the average noxious-evoked EEG activity following a heel lance in a group of term-aged infants. In this study, the magnitude of the noxious-evoked activity was determined by an unblinded analyst using custom-built automated analysis software. The data was first Woody filtered to maximise the correlation between the signal and the template, accounting for individual infant latency variation. The data was Woody filtered by a maximum shift of ± 50 ms in Study 1 and ± 100 ms in Study 2. In both cases, these filter settings ensured that in the control data (with no tactile stimulation) the magnitude of the noxious-evoked activity was significantly greater in response to the first noxious stimulus than in the background EEG activity (Study 1 - experimental noxious stimulus: magnitude of noxious evoked brain activity in background EEG activity = 0.14 ± 0.06; magnitude of noxious evoked brain activity following experimental noxious stimulation = 0.42 ± 0.12; $p=0.022$, two-sided t-test; Study 2 - heel lance stimulation: magnitude of noxious evoked brain activity in background EEG activity = 0.42 ± 0.08; magnitude of noxious evoked brain activity following heel lance stimulation = 1.21 ± 0.17; $p<0.001$, two-sided t-test). The higher Woody filter was used in Study 2 because the 50 ms filter was not sufficient to align the template with the evoked activity, and therefore significant noxious evoked activity following the heel lance was not characterised relative to the background EEG activity. Individual waveforms following heel lance stimulation in all infants are provided in Figure S2C.

Goodness-of-fit of the template of noxious-evoked brain activity was calculated as the Pearson's correlation coefficient of the template and the Woody filtered EEG signal in the time window 400-700 ms after the stimulus.

EMG

EMG signals were filtered 10 – 500 Hz, with a notch filter at 50 Hz (and harmonics). Signals were extracted in 6-second epochs with 2 seconds before the stimulus and rectified. Individual epochs were rejected due to artefact, or if movement occurred in the baseline period. In Study 1, considering the response to the first trial in each block, data from 9 infants in the CT-optimal touch condition, 9 in the CT non-optimal touch condition, and 6 in the no touch condition were rejected. In Study 2, 3 infants from the CT-optimal touch group, and 2 from the no touch control group, were rejected (leaving 13 and 14 infants respectively in the analysis). The magnitude of the reflex withdrawal for each trial was analysed using the root-mean-square (RMS) of the signal. The RMS was calculated in 250 ms windows, and the average RMS across the first second after the stimulus (i.e. across 4 windows) was calculated.

Facial expressions

In Study 2, two blinded trained observers assessed the duration of nasolabial furrow, brow bulge and eye squeeze (according to the Premature Infant Pain Profile-Revised (PIPP-R) [S10] in the 30 seconds after the heel lance retrospectively from the video

recordings. Two infants in the control group were not included in the analysis due to technical difficulties. Infants were defined to have a facial expression change if they exhibited any of these three behaviours during the 30-second period, and the duration of the facial expression response in infants who responded was calculated as the average duration of the three behaviours. Intra-rater (in all infants) and inter-rater (in a subset of 18/30 infants) reliability of facial expression scores were calculated using intra-class correlation, and were 0.97 and 0.92 respectively. Facial expressions were not recorded in Study 1 as the experimental noxious stimuli do not evoke significant responses [S2].

Quantification and Statistical Analysis

Statistical analysis was carried out using R (The R Project for Statistical Computing) and MATLAB (Mathworks, R2017a). The pre-defined primary outcome measure for Study 1 was the comparison of the magnitude of the noxious evoked brain activity between the CT-optimal brush condition and the no-brush condition in response to the 1st experimental stimuli. The magnitude of the noxious evoked brain activity was compared between modalities using linear mixed-effects models (using the nlme R package), with modality set as a fixed effect and individual infants taken as random effects. A p value < 0.05 was considered significant.

Secondary questions addressed whether the CT-non-optimal brush condition reduced the noxious-evoked activity compared with the no-brush condition; whether repeated applications of the CT-optimal brush stimulation reduced the noxious-evoked activity compared with the no-brush condition; and whether the magnitude of the reflex withdrawal activity (recorded using EMG on the biceps femoris of the stimulated leg) was different between the CT-optimal brush condition and the no-brush condition in response to the first experimental stimuli. For all secondary analyses, a p-value of 0.01 (two-sided 1% significance level) was used to indicate statistical significance to adjust for multiple comparisons. Linear mixed-effects models were used, except in the analysis of the magnitude of the reflex withdrawal activity, where the data was skewed and Wilcoxon signed rank tests were used.

Following the observation that there was a significant reduction in the noxious-evoked activity following the first application of the CT-optimal stimulation compared with the control group (primary outcome measure) but not a reduction with repeated application, a post-hoc analysis was conducted to investigate whether the application of the first stimulus in the CT-optimal brush condition evoked significantly lower magnitude noxious-evoked activity compared with each subsequent application of stimulation. The analysis was conducted using a linear mixed-effects model, with trial as a fixed effect and individual infants taken as random effects.

In Study 2, a two-tailed t-test was performed to compare the magnitude of the noxious-evoked brain activity between the two groups. The magnitude of the reflex withdrawal activity, the proportion of infants who exhibited facial expression change and the duration of facial expression change were also compared between each group. As the magnitude of the reflex withdrawal activity and facial expression duration data were

skewed, comparison between groups was performed using the Mann-Whitney U test. Standard error of the median is calculated using 1000 bootstrap samples of the data. Comparisons of each modality with the control group and the background activity were conducted to ensure that the noxious-evoked brain activity was well characterised within each study population.

Acknowledgements

This work was funded by the Wellcome Trust (Senior Research Fellowship, 207457/Z/17/Z).

Supplemental References

S1. Hartley, C., Duff, E.P., Green, G., Mellado, G.S., Worley, A., Rogers, R., and Slater, R. (2017). Nociceptive brain activity as a measure of analgesic efficacy in infants. Science translational medicine *9*, eaah6122.

S2. Hartley, C., Goksan, S., Poorun, R., Brotherhood, K., Mellado, G.S., Moultrie, F., Rogers, R., Adams, E., and Slater, R. (2015). The relationship between nociceptive brain activity, spinal reflex withdrawal and behaviour in newborn infants. Scientific reports *5*, 12519.

S3. Hartley, C., Moultrie, F., Gursul, D., Hoskin, A., Adams, E., Rogers, R., and Slater, R. (2016). Changing balance of spinal cord excitability and nociceptive brain activity in early human development. Current Biology *26*, 1998-2002.

S4. Goksan, S., Hartley, C., Emery, F., Cockrill, N., Poorun, R., Moultrie, F., Rogers, R., Campbell, J., Sanders, M., Adams, E., et al. (2015). fMRI reveals neural activity overlap between adult and infant pain. Elife *4*.

S5. Pawling, R., Cannon, P.R., McGlone, F.P., and Walker, S.C. (2017). C-tactile afferent stimulating touch carries a positive affective value. PloS one *12*, e0173457.

S6. Fairhurst, M.T., Löken, L., and Grossmann, T. (2014). Physiological and behavioral responses reveal 9-month-old infants’ sensitivity to pleasant touch. Psychological science *25*, 1124-1131.

S7. Liljencrantz, J., Strigo, I., Ellingsen, D.M., Kramer, H.H., Lundblad, L.C., Nagi, S.S., Leknes, S., and Olausson, H. (2017). Slow brushing reduces heat pain in humans. Eur J Pain *21*, 1173-1185.

S8. Hartley, C., Poorun, R., Goksan, S., Worley, A., Boyd, S., Rogers, R., Ali, T., and Slater, R. (2014). Noxious stimulation in children receiving general anaesthesia evokes an increase in delta frequency brain activity. Pain *155*, 2368-2376.

S9. Worley, A., Fabrizi, L., Boyd, S., and Slater, R. (2012). Multi-modal pain measurements in infants. Journal of neuroscience methods *205*, 252-257.

S10. Stevens, B.J., Gibbins, S., Yamada, J., Dionne, K., Lee, G., Johnston, C., and Taddio, A. (2014). The premature infant pain profile-revised (PIPP-R): initial validation and feasibility. The Clinical journal of pain *30*, 238-243.
